# Alendronate-associated osteonecrosis of the jaws: A review of the main topics

**DOI:** 10.4317/medoral.19094

**Published:** 2013-08-29

**Authors:** Felipe Paiva-Fonseca, Alan R. Santos-Silva, Ricardo Della-Coletta, Pablo A. Vargas, Márcio A. Lopes

**Affiliations:** 1 DDS, MSc - Piracicaba Dental School - State University of Campinas - UNICAMP, Brazil; 2 DDS, PhD - Piracicaba Dental School - State University of Campinas - UNICAMP, Brazil

## Abstract

Bisphosphonates is a group of inorganic pyrophosphates analogues that suppress bone resorption by inducing osteoclast inactivation, being frequently used for management of diseases affecting bone metabolism, bone metastases and bone tumors. However, since 2003 many cases describing the presence of necrotic bone exposures in the jaws have been described in patients receiving these drugs, what represent a significant complication of bisphosphonates treatment. The overall incidence of bisphosphonate-related osteonecrosis of the jaws is low, ranging from 0.7% to 12%, mainly observed in those patients receiving intravenously treatment. Osteonecrosis of the jaws associated to oral bisphosphonate, particularly alendronate, has also been reported by a number of authors. Considering that alendronate is one of the most used drugs worldwide, specially for treatment of osteoporosis, a better understanding of osteonecrosis of the jaws related to its use and how to manage these patients is extremely important. Therefore, in the current manuscript the authors aim to review the most important topics related to this pathological presentation.

** Key words:**Bisphosphonates, alendronate, bisphosphonate-related osteonecrosis of the jaws, osteonecrosis.

## Introduction

Bisphosphonates (BPs) is a group of analogues of inorganic pyrophosphates that suppress bone resorption by inhibiting farnesyl pyrophosphate synthase enzyme in osteoclasts, thus interfering with geranylgeranylation (attachment of the lipid to regulatory proteins), finally inducing osteoclast inactivation and inhibiting its function and maturation. BPs have been currently considered the first-choice therapy in the management of diseases affecting bone metabolism (osteoporosis and Paget’s disease) and bone metastases (1-4). BPs differ one from another in substitution of the active side chains on their phosphorous-carbon-phosphorous structural backbone. The first generation of BPs (etidronate, clodronate) possesses alkyl or halide side groups, whereas the second generation (pamidronate) contains an amino-terminal group. In 2002 it was authorized the clinical use of the zoledronic acid (zoledronate) that represented the third generation of BPs. Zoledronate contains an imidazole ring group in its side chain and is about 100 times more potent than pamidronate and even stronger than the first generation of BPs (2,5-7).

**Figure 1:**
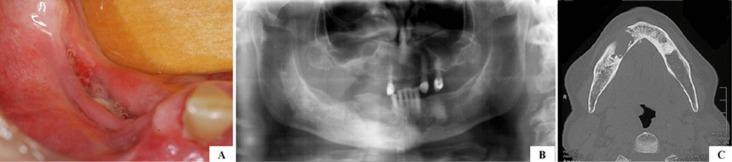
A) Clinical aspect of AONJ presenting an uncommon and extensive osseous involvement well illustrate in B) panoramic radiograph and C) computed tomography exams.

Although BPs are efficient drugs, since 2003 some reports have presented necrotic bone exposures in patients receiving these drugs (Fig. 1), representing a significant complication of BP treatment. The American Association of Oral and Maxillofacial Surgeons (AAOMS) defined the bisphosphonate-related osteonecrosis of the jaws (BRONJ) as the presence of exposed necrotic bone in the maxillofacial region that does not heal within 8 weeks after clinical identification, in a patient currently or previously treated with BPs, who has never undergone radiotherapy to the jaws (8). The overall incidence of BRONJ is low, ranging from 0.7% to 12% in those patients submitted to intravenously treatment (8).

These drugs can also be administered orally and alendronate represents by far the oral nitrogen-containing BP most commonly used to treat osteoporosis, what is well illustrated by the 27 million prescriptions in United States in 2008 (75% for alendronate). With the oral administration, even after only a few weeks of intake, adequate levels of bone resorption inhibition can be observed (1,2,9). However, occurrence of osteonecrosis of the jaws related to alendronate has also been reported by several authors (1,2,6,8).

The aim of the current article is to review key issues concerning alendronate-associated osteonecrosis of the jaws (AONJ), discussing its potential risk factors, treatment options and the awareness of the health care workers and of the patients that are receiving this therapy.

## Material and Methods

-What is the potential risk for alendronate-associated bone necrosis?

Oral BPs are potent osteoclast inhibitors, but they are not highly efficacious in the treatment of malignant osteolytic disease and, therefore, are mostly indicated for the treatment of osteoporosis (10). It is estimated that over 190 million prescriptions for oral BP have been dispensed worldwide and alendronate is by far the most common of the oral BPs prescribed (11).

AONJ has been reported by a number of authors and given the increasing number of persons taking alendronate, a better understanding of the epidemiology of osteonecrosis of the jaws and oral BP therapy is critical (12,13). According to the manufacturer of Fosamax® (commercial name of alendronate) (Merck, Whitehouse Station, NJ, USA), an incidence rate of 0.7 to 1 cases of AONJ per 100,000 patients taking the drug or 170 cases worldwide, was suggested for 2006. The current estimate prevalence has ranged from 0.001% to 0.01% among oral bisphosphonate-treated populations, what is significantly inferior than the incidence observed in patients undergoing intravenous therapy (3,4,12-15). However, Sedghizadeh et al. (14) found an incidence as high as 4% of AONJ by evaluating 208 osteoporotic patients, indicating that this incidence could be higher than the usually reported.

Female patients in the sixth to seventh decade of life have been reported to be more affected by AONJ than males, what may be consequence of the significantly higher incidence of osteoporosis in this group of individuals (11-14). Moreover, patients receiving alendronate due to other pathological entities, such as Paget’s disease, osteogenesis imperfecta and rheumatoid arthritis, have also been reported to develop AONJ (16-19).

Hence, although rarely described if compared to osteonecrosis of the jaws associated to the use of intravenous BPs, AONJ must be considered a potential side-effect of patients under alendronate therapy, most commonly seen in elderly female patients affected by osteoporosis.

-What are the predisposing factors?

Despite the nature of BRONJ has not been well determined and the impossibility of establishing the real risk for developing BRONJ for a certain patient, several risk factors have been connected. Hence, the role of comorbidities in the pathogenesis and prognosis of BRONJ has been suggested and the presence of diseases that compromise blood supply, cellular metabolism, oxygenation, and immune response such as diabetes, hypertension, hypercholesterolemia and obesity, as well as the chronic use of corticosteroids, methotrexate and thalidomide and smoking habit may have a synergistic effect in the initiation and outcome of oral and intravenous BRONJ (9,14,20).

In addition, it is well known that not only the potency of BPs, but also the period of taking and route of administration are important features for determining the risks for osteonecrosis onset. Specifically regarding oral BPs it has been shown that the risk of developing ONJ is closely related to the therapy duration and it seems to be higher after 3 years of treatment (14,21). Lo et al. (12) observed that the prevalence of osteonecrosis was greater among patients receiving oral BPs with more than 4 years of exposure compared with those with less than 4 years of exposure. Similarly, Marx et al. (22) reported an exponential relationship between the size of bone exposure and the duration of oral BP use, observing that all patients who developed osteonecrosis took an oral BP for more than 3 years.

Local variables could also raise the incidence of BRONJ (2,23). Tooth extraction is the event most commonly preceding BRONJ in clinical series and given the available evidences, current guidelines discourage tooth extraction in patients receiving both oral and IV BPs (8,14,21,24,25). Manfredi et al. (20) observed a history of oral surgery at the site affected by osteonecrosis in 58.3% of their patients receiving alendronate therapy and the majority of them reported tooth extraction months before AONJ appearance. Different authors also identified local trauma resulting from ill-fitted dentures as the initiating event in a percentage of their AONJ patients, highlighting this necessity of adequate prosthodontic follow-up for these group of individuals (18,26,27). Moreover, bony outgrowths such as mandibular and palatine tori can be easily traumatized and should be recognized as potential sites at risk for AONJ (21,22,28-30).

Regarding dental implants, the AAOMS divides patients taking oral-BPs into two risk groups. 1) those who have been taking oral-BPs for < 3 years and are directly eligible for implant treatment and 2) those who have been taking oral-BPs for > 3 years or for < 3 years but simultaneously taking corticosteroids. For this second group a BP drug interruption 3 months before and 3 months after implant therapy was recommended if systemic health of the patient allows (21).

In terms of implant failure, Jeffcoat (31) reported 100% success rate for 102 implants with no clinical evidence of infection, pain or bone necrosis in patients receiving alendronate for a mean duration of 3 years. Marx et al. (22) observed that only 6.7% of their sample receiving oral BPs developed osteonecrosis as a result of implant placement. Similarly, Martin et al.(32) observed that only 2.7% of their patients receiving oral BPs (589 patients) exhibited implants failures, whereas Grant et al. (33) by evaluating 115 patients who had received oral BP therapy and dental implants, observed no evidence of BRONJ in any of the patients evaluated. Fugazzotto et al. (34) retrospectively evaluating 61 osteoporotic female patients under oral BP therapy observed that only one patient demonstrated a small tissue dehiscence at the 1-week postoperative examination, stating that oral BPs use for a mean period of 3.3 years would not be a contributing factor to the development of osteonecrosis following implant placement. Hence, taken these results together, it can be assumed that implant success for patients receiving oral BP therapy is similar to those not receiving oral BP therapy.

With the purpose of identifying which patients had a greater risk of BRONJ, it has been proposed the usefulness of determining serum CTX (telomere C-terminal of collagen 1), a biologic index to measure bone remodeling and bone resorption, pointing out that those patients who had taken oral BPs for more than 3 years and who had CTX below 150 pg/ml would be at greater risk of developing BRONJ during dental surgery (22,35). Kwon et al. (36) correlated the staging of osteonecrosis with serum CTX in 18 osteoporotic patients receiving oral BPs treatment, the authors found a significant correlation between the disease severity and the risk assessment using serum CTX. However, Fleisher et al. (37) evaluating 26 patients receiving IV or oral BPs with CTX levels < 150 pg/ml that underwent teeth extractions or surgical procedures for treatment of BRONJ, observed that all individuals healed successfully after surgical managements, emphasizing that healing for these patients can occur even in the presence of low serum CTX levels and that other clinical parameters, such as periodontal changes, could be more predictable of BRONJ development.

In summary, although some reports indicate that AONJ of the jaws can develop spontaneously without a clear cause (5,19,22), numerous risk factors are currently effectively associated with an increased AONJ rates especially the duration of therapy and the history of tooth extraction. However, in contrast to the contraindicative advertisements of all guidelines concerning implant placement in patients receiving IV-BPs, patients treated with alendronate do not seem to present increased rates of implant failures if compared to individuals who have never undergone alendronate therapy. Moreover, although further studies are necessary to confirm the clinical efficacy of CTX serum levels in predicting clinical behavior and risk of developing osteonecrosis in patients taking BPs, this seems to be a useful laboratorial parameter for managing patients under oral BPs treatment.

-How to treat bone necrosis? 

The management of patients with BRONJ remains extremely difficult, with numerous recommendations being proposed and controversially discussed, but no consensus on a standard of care has been reached. In addition, there is no agreement on a surgical versus nonsurgical approach to therapy, especially because most of the relevant literature reports heterogeneous case studies or retrospective analyses that are difficult to be compared (23,28).

Surgical debridement with the goal of covering the exposed bone has not been completely effective. The uncertain outcomes of surgical treatment probably stem from the inability of surgically remove the necrotic bone to a safe margin with viable bleeding bone. In addition, surgery may create a bone wound that will not heal because of the BP therapy itself, originating a secondary osteonecrosis from the surgical margins (10,38). Therefore, contradictory results have been described by different authors regarding the efficacy of surgical approaches in BRONJ treatment.

Specifically concerning oral BPs, it is known that they present an inferior potency than their intravenous counterpart, since orally administered BPs allow less than 1% of drug absorption. It has been observed that oral bisphosphonate-related ONJ is generally less severe and it is more responsive to a more conservative treatment than IV bisphosphonate-induced osteonecrosis. In addition, it seems that ONJ oral bisphosphonate-related correlates well to the serum CTX levels, therefore, becoming more predictable (12,22,29). Hence, it has been suggested that for patients who present with exposed bone due to an oral BP, the drug should be discontinued by the prescribing physician if possible and a CTX test be required. If the exposed bone is painless, a conservative approach with 0.12% chlorhexidine is initially sufficient. However, if the patient reports pain or if clinical evidence of infection is present, antibiotic therapy should be provided in addition to the 0.12% chlorhexidine. Surgery with local debridement should be reserved for cases refractory to these initial conservative approaches (21,22).

**Figure 2:**
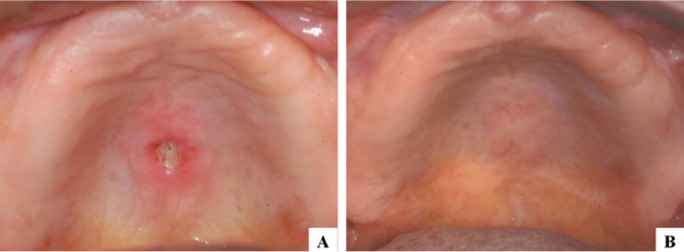
A) AONJ on the palate associated with trauma caused by prosthesis. B) The lesion exhibited complete resolution following conservative treatment.

**Figure 3:**
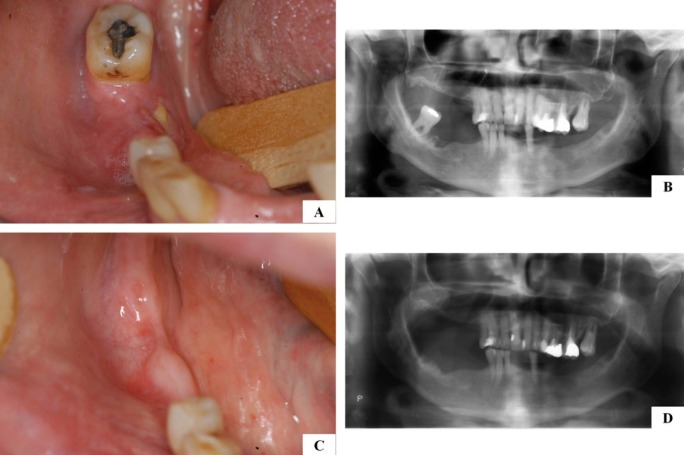
A, B) Clinical and radiographic aspects of AONJ after tooth extraction. C, D) Complete resolution after conservative removal of the necrotic bone.

In summary, although innumerous guidelines have been suggested by different groups in the last years regarding BRONJ treatment, no consensus has been obtained. Fortunately, it has been noted that AONJ may represent a significantly less aggressive and more predictable condition if compared to IV BPs-ONJ, arguing for a more conservative management with improved oral hygiene and local and systemic antibiotics, reserving surgical approaches for refractory situations. Figures 2,3 illustrate two patients who developed AONJ and had complete resolution.

-Interrupt drug use may prevent bone necrosis?

Additionally to the absence of a well established therapeutic approach in cases of BRONJ, the uncertainties concerning the influence of BP treatment discontinuation in the outcomes of the patients undergoing BRONJ treatment, remains highly obscure. It is known that nitrogen-containing BPs are not metabolized and that 50% of it are secreted in the urine unchanged and the rest bind to bone, being slowly released into the circulation. As a consequence, their half-life in the bone could be as long as 10 years. Therefore, it is thought that the actions of BPs may continue for all this time even after they have been discontinued (3,14,23,28,38).

When evaluating 25 osteoporotic patients treated with oral BP, Manfredi et al. (20) did not observe differences in the healing process between individuals who suspended treatment and those who did not. However, other studies have shown that the cessation of oral or IV BP treatment before dental surgical approaches would significantly favor an improvement in patients’ outcome and reduce the risk for BRONJ, probably because the newly formed bone would be unable to absorb BPs (23). Marx et al. (22) observed that 6-month drug holiday showed a direct and significant improvement in the CTX values in patients receiving oral BP therapy, demonstrating a clinical bone recovery and important response to a drug discontinuation.

Thus, recent reports are in favor of giving to the patient a drug holiday before surgical procedures is performed especially for individuals receiving oral BPs. However, the decision to suspend BP treatment should be taken by the prescribing physician rather than the dentist, and before considering the discontinuation of the oral BP therapy for a patient with osteoporosis, the very limited risk of AONJ must be weighed against the positive effects on health of oral BP use (9,20).

-How aware are the patients and health care workers about the side-effects?

The increasing frequency of new cases being described in the literature and the appearance of consensus guidelines on BRONJ prompted some researchers to determine the level of knowledge of patients, dentists and dental students concerning BPs and osteonecrosis. Lópes-Jornet et al. (35), Migliorati et al. (15) and Bauer et al. (3) observed that in all groups only a minority knew adequate information concerning the use of BPs, suggesting that greater educational efforts should be made to promote knowledge of this pathology at both undergraduate and postgraduate levels, and that the majority of the patients receiving oral or IV BPs are not adequately informed about possible adverse effects of their BP therapy.  Table 1 shows the most important topics related to AONJ.

**Table 1:**
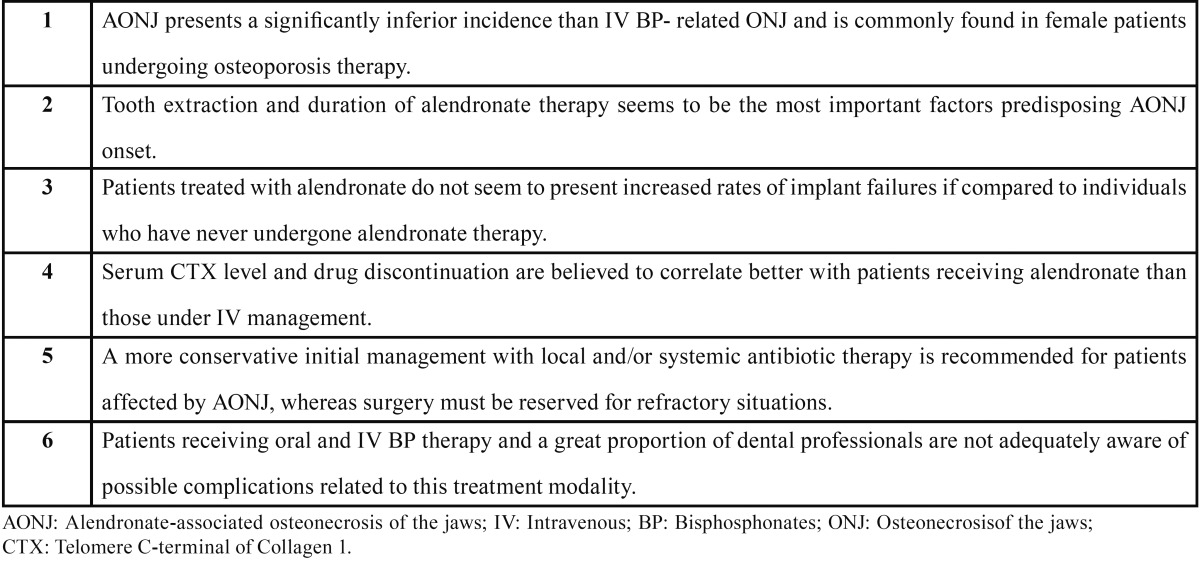
Summary of the most important topics related to AONJ.

## Conclusions

Patients under oral bisphosphonate use are of lower risk for bone necrosis of jaw, this side-effect is generally less severe and it is more responsive to treatment comparing to ONJ in patients taking intravenous formulations. Although relatively uncommon, dental extraction is the most common risk factor for ONJ. Drug discontinuation and serum CTX levels may be useful to prevent ONJ. Finally, physicians, dentist and patients should be aware of this possible side-effect of oral BP therapy, since the knowledge and dental preventive measures can significantly reduce the risk of developing this condition.
